# A combination of 3-D discrete wavelet transform and 3-D local binary pattern for classification of mild cognitive impairment

**DOI:** 10.1186/s12911-020-1055-x

**Published:** 2020-02-21

**Authors:** Harsh Bhasin, Ramesh Kumar Agrawal, For Alzheimer’s Disease Neuroimaging Initiative

**Affiliations:** School of Computer and Systems Sciences, Jawaharlal Nehru University, New Delhi, India

**Keywords:** Mild cognitive impairments, Machine learning, 3D discrete wavelet transform, 3D local binary pattern, Magnetic resonance imaging

## Abstract

**Background:**

The detection of Alzheimer’s Disease (AD) in its formative stages, especially in Mild Cognitive Impairments (MCI), has the potential of helping the clinicians in understanding the condition. The literature review shows that the classification of MCI-converts and MCI-non-converts has not been explored profusely and the maximum classification accuracy reported is rather low. Thus, this paper proposes a Machine Learning approach for classifying patients of MCI into two groups one who converted to AD and the others who are not diagnosed with any signs of AD. The proposed algorithm is also used to distinguish MCI patients from controls (CN). This work uses the Structural Magnetic Resonance Imaging data.

**Methods:**

This work proposes a 3-D variant of Local Binary Pattern (LBP), called LBP-20 for extracting features. The method has been compared with 3D-Discrete Wavelet Transform (3D-DWT). Subsequently, a combination of 3D-DWT and LBP-20 has been used for extracting features. The relevant features are selected using the Fisher Discriminant Ratio (FDR) and finally the classification has been carried out using the Support Vector Machine.

**Results:**

The combination of 3D-DWT with LBP-20 results in a maximum accuracy of 88.77. Similarly, the proposed combination of methods is also applied to distinguish MCI from CN. The proposed method results in the classification accuracy of 90.31 in this data.

**Conclusion:**

The proposed combination is able to extract relevant distribution of microstructures from each component, obtained with the use of DWT and thereby improving the classification accuracy. Moreover, the number of features used for classification is significantly less as compared to those obtained by 3D-DWT. The performance of the proposed method is measured in terms of accuracy, specificity and sensitivity and is found superior in comparison to the existing methods. Thus, the proposed method may contribute to effective diagnosis of MCI and may prove advantageous in clinical settings.

## Background

Alzheimer’s Disease (AD) is one of the most common neuro-degenerative disease in elderly people [1]. According to the National Institute of Ageing, though Alzheimer’s is incurable, it is not intractable [2]. Medicines are available for treating the symptoms and the established strategies can be employed to enhance the general brain and mental conditions. However, the treatment is effective only in the case of early detection. Mild Cognitive Impairment (MCI) is a prodromal stage of dementia and can be considered as a transitional phase from the likely cognitive decline of normal aging to the graver decline of dementia [3]. This makes the diagnosis of MCI predominantly vital as appropriate steps can be taken to slow down the progression.

MCI causes a slight but noticeable and measurable decline in cognitive abilities, including memory and thinking skills [4]. As per Ward [5], the Annual Conversion Rates, from MCI to AD ranged from 7.5 to 16.5 per person-year. Researchers have identified the following changes in the autopsy studies of people suffering from MCI [6]:

- Abnormal clusters of beta-amyloid protein (plaques)
- Microscopic protein clumps of tau characteristic of AD (tangles)
- Lewy bodies, which are microscopic clumps of another protein associated with forms of dementia like AD and
- Small strokes or reduced blood flow through brain blood vessels.

MCI can be detected by either clinical tests or by brain scans. A medical professional determines the presence or absence of MCI by evaluating a person’s cognitive and behavioural changes and by using professional judgement about the possible causes and severity of the symptoms [6]. Some of the common clinical tests used for the detection of MCI are Mini Mental State Examination, Clock Test, Logical Memory, Rey Auditory Verbal Learning Test, Digit Span Category Fluency Tests, Trail Making Test A-B, Boston Naming Test, American National Adult Reading Test, Alzheimer’s Disease Assessment Scale-Cognitive Behaviour, Geriatric Depression Scale and Functional Assessment Questionnaire [7, 8]. In recent years, the veracity of the brain imaging techniques has been used for the classification of

- MCI and controls and
- MCI-C and MCI-NC.

Imaging modalities like Functional Magnetic Resonance Imaging (fMRI) [9, 10], Positron Emission Tomography (PET) [11], structural Magnetic Resonance Imaging (s-MRI) [12–26], etc. have been used to diagnose MCI.

Manual assessment of MCI requires more time, resources and expertise, which is highly inconvenient and costly to the patient. In the past two decades, detection of MCI has drawn the attention of Image Processing (IP) and Machine Learning (ML) community. ML based methods can be used to distinguish MCI patients from AD patients or controls. These methods require lesser manual intervention of experts and may be less costly. Moreover, these techniques provide better visualization of the huge data compared to manual methods.

It was found, from research works [12–24], that the classification of i) MCI-C and MCI-NC and ii) MCI and CN require immediate attention owing to two reasons: i) The above classes have not been explored copiously ii) The maximum classification accuracy reported is rather low [12–24]. This calls for the application of pertinent feature extraction and feature selection methods which can improve the performance.

The s-MRI can be used to access the structural changes in the brain associated with MCI. Researchers have used cortical atrophy for diagnosing MCI [27]. Moreover, the regions of the brain namely hippocampus, amygdala and ehorhinal cortex have found to be important in the diagnosis of MCI [28]. Researchers [27–29] have explored the Regions of Interest (ROIs) based analysis for automatic MCI diagnosis. These ROIs have been determined either by predefinition or by adaptive parcellation. These methods can be segregated into single ROI methods and multiple ROIs methods. The hippocampal volume was used to discriminate MCI and NC patients by Chupin [15]. The combination of hippocampus features and cerebrospinal fluid (CSF) volume was used for this task by Ahmed et al. [23]. Magnin et al. [26] used 90 features to represent 90 ROIs of the whole brain, where each feature describes relative weight of GM compared to WM and CSF. These approaches miss out the effect of the other regions on the disease and also subdue the fact that the regions of the brain are interconnected.

Texture classification acts like a significant protagonist in the bids of computer vision like image retrieval, video retrieval, and medical diagnosis [30]. In order to carry out texture classification, it is essential to extract good features from an image to distinguish diverse textures. Texture analysis methods like Gray Level Co-occurrence Matrices (GLCM) [31, 32], Discrete Wavelet Transform (DWT) [33], Local Binary Pattern (LBP) [34] etc. have been used in medical image analysis. GLCM measures the average degree correlation between pairs of pixels in different aspects [32]. The discrimination capabilities of GLCM depends on the choice of the separation distance between pixels, which is difficult to ascertain. The Wavelet Transform (WT) allows localization in both spatial and local transients like surfaces in 3D volumes, which help in apprehending finer minutiae of brain MRI data present in different directions. However, the number of features obtained by the DWT is huge. LBP, another popular feature extraction technique, can be used to gauge the statistical and structural information to represent the image [34]. It captures the underlying distribution of various microstructures like edges etc. [35–37]. Moreover, it also represents original data with lesser number of features. However, it does not retain the spatial distribution of different patterns present in the image.

In this paper, in order to generate a rich representation of anatomical structures, which will be more discriminative to separate different groups of subjects, a combination of 3D-DWT and a variant of 3D-LBP is proposed. First level decomposition of an MRI using 3D-DWT provides one approximate and seven detailed components. Each detailed component captures different orientation of micro-structures. However, the number of features obtained from the seven detailed components is large. Each of these seven detailed components is represented compactly using 3D-LBP.

The 3D-LBP with 18 neighbours results in 262,144 features. Further reduction in the number of features can be done by applying the rotation invariant and uniform variants of the LBP. We have investigated i) basic 3D-LBP, ii) rotation invariant 3D-LBP and iii) uniform 3D-LBP and out of these the one which gives the best result has been clubbed with 3D-DWT. To the best of our knowledge, no research work carried out till date has applied a combination of 3D-DWT and 3D-LBP on the s-MRI data to distinguish MCI-C from MCI-NC and MCI from CN. Further, Fisher Discriminant ratio (FDR) is applied to determine a set of relevant features and the well-known Support Vector Machine is used to develop a decision model. The performance of the proposed method is compared with existing methods on a publicly available ADNI data.

## Availability of data and materials

### ADNI database

The datasets supporting the conclusions of this article are available in the Alzheimer’s Disease Neuroimaging Initiative (ADNI) repository, Data used in the preparation of this article were obtained from the ADNI database (adni.loni.usc.edu). The ADNI was launched in 2003 as a public-private partnership, led by Principal Investigator Michael W. Weiner, MD. The primary goal of ADNI has been to test whether Magnetic Resonance Imaging (MRI), PET, other biological markers, and clinical and neuropsychological assessment can be combined to measure the progression of MCI and early AD.

### Retrieval of data

The ADNI database was queried for controls (CN), those converted to AD (MCI-C) and those not converted to AD (MCI-NC). The protocol of data selection and image acquisition of the subjects takes into consideration age matching, appropriate number of slices, required parameters etc. and has been adopted from paper [38]. However, the data of all the CN subjects was not available and hence more CN subjects were selected from the database. This study uses 75 MCI-C, 89 CN and 112 MCI-NC processed NIFTI images of patients. The MCI-NC patients ranged between the ages 56 and 88. The controls were of the ages between 63 and 90 and MCI-C patients ranged between the ages 55 and 87. All the patents had Mini Mental State Examination score between 18 and 27 and a CDR of 0.5 or 1. The T1 weighted s-MRI images collected had the following field strength: 1.5 Tesla, TE = 3.6099 ms, TE = 3000 ms. Table 1 shows the relevant data of the patients.

**Table 1:** Demographic and Clinical Information of the subjects

Group	MCI-NC (*n* = 112)	MCI-C (*n* = 75)	CN (*n* = 89)
Female/ Male	51/61	33/42	45/44
Age (Mean ± SD)	74.83 ± 7.34	74.69 ± 7.28	75.21 ± 5.13

### Pre-processing

In the literature, Structural Magnetic Resonance Imaging (s-MRI) has been widely used to detect MCI. However, before extracting features from an image, pre-processing is required. For this, Statistical Parametric Mapping (SPM) is used [39].

The pre-processing of the s-MRI images includes the following: a) Slice time correction, which is required if the temporal dynamics of evoked responses are important. This was performed as it may improve the performance; b) Head motion correction, which is referred to as realignment; c) Spatial Normalization, which is the co-registration with the standard MNI template in order to overcome brain shape variability; d) Special smoothening, in which the weighted average of the neighbouring voxels is found and the intensity value of a voxel is replaced by it and e) Tissue segmentation, which is the segregation of brain tissues into three tissue classes namely gray matter, white matter and cerebro-spinal fluid. In the literature, it has been found that gray matter atrophy is responsible for Mild Cognitive Impairments [40, 41]. For this reason, gray matter is used for building a ML based model to diagnose MCI. The most common method to measure differences in local concentrations of brain tissue is Voxel Based Morphometry (VBM). In VBM a voxel-wise comparison of the local concentration of gray matter between the two groups of subject is carried out [42]. However, in VBM also, pre-processing is required which includes registration to a standard template, followed by smoothening and segmentation.

## Methods

### 3D-discrete wavelet transform

The Fourier Transform (FT) is commonly used to determine the frequency spectrum of a signal, for better analysis of the signal. However, in the case of a non-stationary signal, FT is not of much use. For signals where time localization of spectral components is needed, one solution is to adopt Short-Time-Fourier-Transform (STFT) to get frequency components of local time intervals of fixed duration. However, in the case of signals having non-periodic fast-transitions (i.e. high frequency content for short duration), wavelet transform (WT) is suggested to be a better option in literature [43]. The WT analyses a signal at different frequencies with different resolutions, which makes it to be an excellent tool for the analysis of transient signals.

WT can be categorized on the basis of orthogonal property of wavelet into Continuous wavelet transform (CWT), which uses non-orthogonal wavelet and Discrete Wavelet Transform (DWT) which uses orthogonal wavelet. The CWT is often used to characterize singularities in functions, but disadvantages like infinite number of wavelets, redundancy, no analytical solutions for most of the functions in CWT, etc. make it difficult to use [43]. Hence, DWT is commonly used in literature.

There are two filters involved in the analysis bank of DWT, one is the wavelet (detailed) filter, and the other is the scaling (averaging) filter. Wavelet expansion of a discrete function *f*(*x*) in terms of wavelet *ψ*(*x*) and scaling function *τ*(*x*) is defined as follows [43]:

1 $$ f(x)=\frac{1}{\sqrt{A}}\sum \limits_a{W}_{\tau}\left(P,a\right){\tau}_{P,a}(x)+\frac{1}{\sqrt{A}}\sum \limits_{p=1}^P\sum \limits_a{W}_{\uppsi}\left(p,a\right){\psi}_{p,a}(x) $$

where $\frac{1}{\sqrt{A}}$ is normalizing factor, P is the decomposition level, *ψ*_*p*, *a*_(*x*) are detailed or wavelet coefficients and *τ*_*P*, *a*_(*x*) are averaging or scaling coefficients are discrete functions in and where $a=\left\{\ 0,1,2,\dots, \frac{A}{2^p}-1\right\}$ [44]. The scaling and detailed coefficients are computed as:

2 $$ {W}_{\tau}\left(P,a\right)=\frac{1}{\sqrt{A}}\sum \limits_{x=0}^{A-1}f(x){\overset{\sim }{\tau}}_{\left(P,a\right)}(x) $$

3 $$ {W}_{\psi}\left(p,a\right)=\frac{1}{\sqrt{A}}\sum \limits_{x=0}^{A-1}f(x){\overset{\sim }{\psi}}_{\left(p,a\right)}(x) $$

1D DWT can be extended to 3D DWT for 3D brain volumes. In 3D DWT [44], we have one 3D approximate coefficient (scaling function) *τ*(*a*, *b*, *c*) and seven 3D detailed coefficients *ψ*^*i*^(*l*, *m*, *n*), where *i* ∈ {1, 2, …, 7}. The function *τ*(*a*, *b*, *c*) in 3-D, is the product of *τ*(*a*), *τ*(*b*) *and τ*(*c*). Also, each *ψ*^i^(a, b, c) is the product of all seven possible combinations of 1-D *τ* and *ψ*, with at least one *ψ*. The above functions help us to find ${W}_{\uppsi}^i\left(p,a,b,c\right)$. The functions have been defined as follows [44]:

4 $$ \tau \left(a,b,c\right)=\tau (a)\tau (b)\tau (c) $$

5 $$ {\psi}^1\left(a,b,c\right)=\psi (a)\tau (b)\tau (c) $$

6 $$ {\psi}^2\left(a,b,c\right)=\tau (a)\psi (b)\tau (c) $$

7 $$ {\psi}^3\left(a,b,c\right)=\psi (a)\psi (b)\tau (c) $$

8 $$ {\psi}^4\left(a,b,c\right)=\tau (a)\tau (b)\psi (c) $$

9 $$ {\psi}^5\left(a,b,c\right)=\psi (a)\tau (b)\psi (c) $$

10 $$ {\psi}^6\left(a,b,c\right)=\tau (a)\psi (b)\psi (c) $$

11 $$ {\psi}^7\left(a,b,c\right)=\psi (a)\psi (b)\psi (c) $$

Wavelet expansion of 3D image volume of size *A* × *B* × *C* can be expressed as:

12 $$ f\left(l,m,n\right)=\frac{1}{\sqrt{ABC}}\sum \limits_a\sum \limits_b\sum \limits_c{W}_{\tau}\left(P,a,b,c\right){\tau}_{P,a,b,c}\left(l,m,n\right)+\frac{1}{\sqrt{ABC}}\sum \limits_{i=1}^7\sum \limits_{p=1}^P\sum \limits_a\sum \limits_b\sum \limits_c{W}_{\uppsi}^i\left(p,a,b,c\right){\psi}_{p,a,b,c}^i\left(l,m,n\right) $$

where

13 $$ {W}_{\tau}\left(P,a,b,c\right)=\frac{1}{\sqrt{ABC}}\sum \limits_{a=0}^{a-1}\sum \limits_{b=0}^{b-1}\sum \limits_{c=0}^{c-1}f\left(a,b,c\right){\overset{\sim }{\tau}}_{P,a,b,c}\left(a,b,c\right) $$

14 $$ {W}_{\uppsi}^i\left(p,a,b,c\right)=\frac{1}{\sqrt{ABC}}\sum \limits_{a=0}^{a-1}\sum \limits_{b=0}^{b-1}\sum \limits_{c=0}^{c-1}f\left(a,b,c\right){\overset{\sim }{\psi}}_{p,a,b,c}^i\left(a,b,c\right),i=\left\{1,2,\dots, 7\right\}, $$

15 $$ {\tau}_{P,a,b,c}\left(l,m,n\right)={2}^{\frac{P}{2}}\tau \left({2}^Pl-a,{2}^Pm-b,{2}^Pn-c\right),\mathrm{and} $$

This study uses the ‘db2’ wavelet function.

### Local binary pattern and its variants

Local Binary Pattern (LBP) is a common feature extraction technique [35]. The technique can be used to gauge the statistical and structural information [35]. The LBP values capture the underlying distribution of various microstructures like edges etc. The LBP value of each pixel is calculated by comparing the pixel value with the intensity of its neighbours using the following formula.

16 $$ {LBP}_{Q,R}=\sum \limits_{i=1}^Q{2}^{i-1}\times \xi \left({I}_Q-{I}_{center}\right) $$

where

17 $$ \xi (x)=\Big\{{\displaystyle \begin{array}{c}1, ifx\ge 0\\ {}0, ifx<0\end{array}}\operatorname{} $$

Here, Q is the total number of neighbours and R is the radius from the central pixel, *I*_*center*_. After the computation of LBP value for each pixel, a histogram of 2^*Q*^ bins is obtained.

The 2-dimensional LBP can be extended to 3-dimension as follows. For each voxel, its intensity is compared with the intensity of the neighbouring voxels. The 18 neighbouring voxels present at the Axial, Coronal, Sagittal and diagonal planes have been considered.

This work considers 18 neighbours and threshold their intensity with respect to the central one, *I*_*center*_.

18 $$ {LBP}_{Q,R}=\sum \limits_{i=1}^{18}{2}^{i-1}\times f\left({I}_Q-{I}_{center}\right) $$

where

19 $$ f(x)=\left\{\begin{array}{c}1, if\ x\ge 0\\ {}0, if\ x<0\end{array}\right. $$

The above technique results in a histogram with 262,144 bins. This method henceforth would be referred to as the LBP-3D method.

It may be further noted that the number of bins in the histogram, so obtained, is humungous. This can be further reduced by applying the uniform and rotation invariant methods in 3D. The Uniform LBP is one that contains at most two ‘0 to 1’ or ‘1 to 0’ transitions [35, 36]. In case of an n-bit binary number (n-neighbourhood), there are *n* × (*n* − 1) + 3 uniform binary patterns. The corresponding histogram of a uniform LBP would therefore contain a lesser number of bins as compared to the conventional LBP, which contain 2^*n*^ bins. In this work, 18 neighbourhoods have been used, so by the application of uniform LBP, the number of features is reduced to 309. This method would be henceforth referred to as LBP-309.

The uniform-rotation-invariant LBP considers a pattern and all the patterns obtained by shifting the given pattern one bit to the right till the same pattern is obtained [35]. For an n-bit number, the uniform-rotation-invariant LBP has (*n* + 2) bins thus reducing the number of features to a great extent. For example, in case of 18 neighbourhoods in 3-D, the pattern can be described as a 18-bit number and the number of features to represent each slice 20 in the case of the rotation-invariant version. This method would henceforth be referred to as LBP-20.

### 3D DWT + LBP-20

The experiments bring forth the point that the performance of LBP-20 exceeds the rest of the variants in terms of specificity, accuracy and sensitivity. Moreover, the number of features in LBP-20 is despondently low. For this reason, this work proposes a combination of 3D-DWT and LBP-20 for extracting features. This method would be henceforth referred to as 3D-DWT + LBP-20. Feature Selection.

Some features extracted in brain imaging may be redundant or noisy and even negatively affect the performance of the decision model. This makes the feature selection an indispensable step before performance classification. Fisher Discriminant Ratio (FDR) is a simple and effective method which measures the discrimination power for a given feature between data of two different classes [45]. The FDR score of the i^th^ feature is calculated as follows:

20 $$ FDR\ (i)={\left({m}_1^i-{\mathrm{m}}_2^{\mathrm{i}}\right)}^2/\left({\left({\upsigma}_1^{\mathrm{i}}\right)}^2+{\left({\upsigma}_2^{\mathrm{i}}\right)}^2\right) $$

Where, ${m}_1^i$ is the mean of samples, of the i^th^ feature, that belong to the first class; ${m}_2^i$ is the mean of samples, of the i^th^ feature, that belong to the second class, ${\sigma}_1^i$ is the standard deviation of samples, of the i^th^ feature, that belong to the first class; ${\sigma}_2^i$ is the standard deviation of samples, of the i^th^ feature, that belong to the second class.

Feature with a higher value is considered to be more relevant. Hence, the calculation of the FDR values of features is followed by the arrangement of the features in descending order of FDR values.

### Classification and evaluation

For a two-class problem, the Support Vector Machine crafts a hyperplane, which separates the data points of the two classes by maximum margin [46]. For the purpose of classification, linear kernel is used. To evaluate the performance of the proposed system, accuracy (ACC), sensitivity (SEN), and specificity (SPE) are used, which are calculated as follows:

21 $$ ACC=\frac{TP+ TN}{TP+ TN+ FP+ FN} $$

22 $$ SENS=\frac{TP}{TP+ FN} $$

23 $$ SPEC=\frac{TN}{TN+ FP} $$

where FP, FN, TP and TN and are the number of false positive samples, false negative samples, true positive samples and true negative samples and respectively.

In order to calculate the performance of the proposed decision system and to set the system parameters, nested 10-fold validation scheme is used. In this scheme, the data is divided randomly into 10 equal sized subsets. In each fold 9 subsets are used for training and validation and one subset is used for testing. This process is repeated 10 times and each subset is used exactly once for testing. This constitutes outer folds. In each outer fold, 10 inner fold are made such that the training and validation data is further divided into 10 equal subsets. In each inner fold 9 subsets are used for training data and one for validation. The experimental results of the inner folds are used for setting the system parameters.

In the experiment, for each feature the average accuracy was found over the outer 10 CV-Fold. The variation of this average accuracy with the number of features was then noted. The number of features for which the performance is best is then reported. The average and the standard deviation are then reported as the performance of the proposed system.

**Fig. 1:**
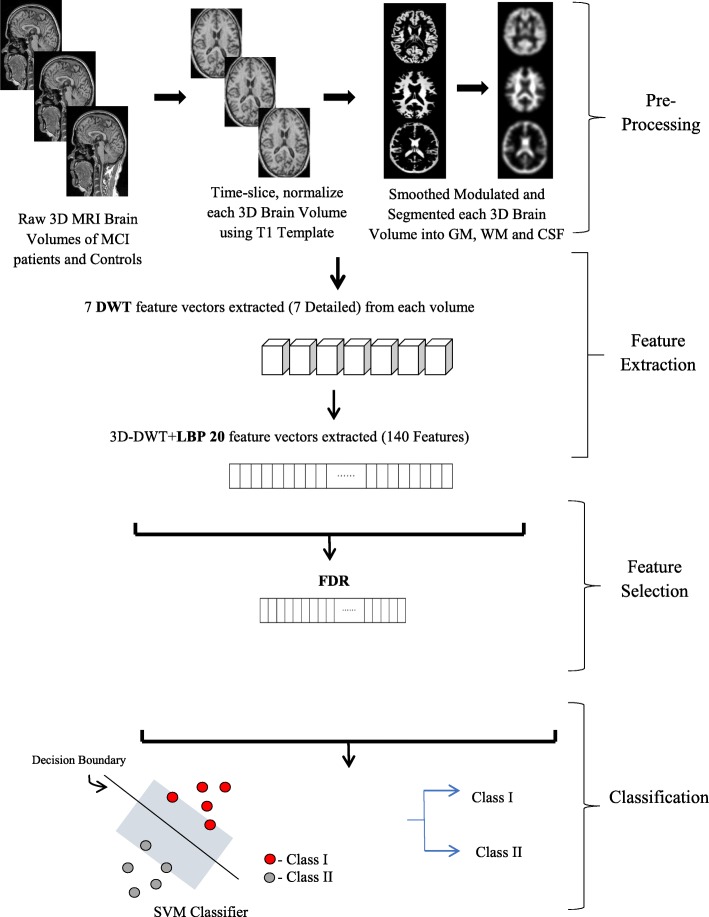
Flowchart of the training phase of 3D-DWT-LBP-20

The procedure for the proposed framework is summarized as follows. It is also depicted in Fig. 1.

#### Diagnosis of MCI

For the given data

1. Divide the data into train and test set.
2. Perform the following computation for each MRI volume of the train set:
  - Extract 7 detailed components from each MRI using 3D-DWT.
  - Obtain features from each of the 7 detailed components using 3D- LBP-20.
  - Concatenate the features obtained from the 7 volumes to obtain 140 features.
3. Apply FDR to order the features obtained from the step 2 in terms of their relevance (decreasing values of their FDR values). The indices so obtained would be used in testing phase.
4. Train the model using the train set.
5. For the test set apply all the steps of 2. Use the indices obtained in step 3) to represent the MRI volume. Obtain accuracy, specificity and sensitivity of the proposed method.

## Results

Firstly, the proposed pipeline is applied to distinguish MCI-C from MCI-NC. The features from the pre-processed data are extracted through the LBP-3D method which results in 262,144 features. The features are placed in the descending order of their FDR values and are included incrementally in order of their relevance to develop a decision model. This work uses nested cross validation. It may be noted that the maximum accuracy of 0.7644 is. The same procedure is used with LBP-309 in which the maximum accuracy of 0.8361 is obtained. In the case of LBP-20, the maximum accuracy of 0.8432 is obtained. The application of 3D-DWT results in the maximum accuracy of 0.8574. Based on these observations 3D-DWT is combined with LBP-20, which results in a maximum accuracy of 0.0.8877. Similarly, the proposed combination of methods is also applied to distinguish MCI from CN data. Results similar to those obtained in MCI-C and MCI-NC data are observed.. The summarized results are shown in Table 2.

**Table 2:** Comparison of performance of various methods to distinguish MCI-C from MCI-NC and MCI from CN

Dataset	Model	Original Number of Features	Accuracy	Specificity	Sensitivity	Average Number of Features
MCI-C vs. MCI-NC	3D DWT	1,359,872	0.8574 ± 0.0073	0.8564 ± 0.0068	0.8555 ± 0.0099	271.8 ± 79.35
	LBP-3D	262,144	0.7644 ± 0.0395	0.7497 ± 0.0291	0.8100 ± 0.0237	232.6 ± 29.28
	LBP-309	309	0.8361 ± 0.0209	0.8453 ± 0.0264	0.8363 ± 0.0238	194.5 ± 59.68
	LBP-20	20	0.8432 ± 0.0131	0.8416 ± 0.0093	0.8411 ± 0.0132	16 ± 2.49
	3D DWT + LBP-20	140	0.8877 ± 0.0167	0.8916 ± 0.0216	0.9016 ± 0.0054	15.3 ± 2.40
MCI vs. CN	3D DWT	1,359,872	0.8834 ± 0.0072	0.8846 ± 0.0064	0.8802 ± 0.0072	235.6 ± 95.01
	LBP-3D	262,144	0.7763 ± 0.0267	0.7805 ± 0.0331	0.7900 ± 0.0342	230.4 ± 3 8.29
	LBP-309	309	0.8791 ± 0.0134	0.8780 ± 0.0167	0.8631 ± 0.0158	188 ± 67.42
	LBP-20	20	0.8847 ± 0.0086	0.8699 ± 0.0137	0.8575 ± 0.0115	15.1 ± 2.33
	3D DWT + LBP-20	140	0.9031 ± 0.0137	0.9015 ± 0.0168	0.9022 ± 0.0134	16.2 ± 1.39

## Discussion

From the results presented in Table 2, the following can be inferred:

- The combination of 3D-DWT with LBP20 after a certain number of features gives better performance than individual feature extraction methods such as 3D-DWT, LBP-3D, LBP-309, LBP-20. The combination provides maximum accuracy of i) 0.8877in the case of MCI-C vs MCI-NC and ii) 0.9031 MCI vs CN.
- LBP-3D gives poorest performance amongst the variants of LBP and 3D DWT.
- LBP-20 gives the best performance amongst the variants of LBP and utilize only 20 features for this.
- For each feature extraction method, the performance improves with the increase in number of features initially and becomes almost stable thereafter.
- On applying the proposed method, the specificity of 0.9016 and the sensitivity of 0.8916 is obtained in the case of MCI-C vs MCI-NC and the specificity of 0.9022 and the sensitivity of 0.9015 is obtained in the case of MCI vs CN.

The comparison of the accuracy, specificity and sensitivity of the proposed model with the existing models is presented in Table 3. It can be observed from the table that the proposed method performs well not only in terms of classification accuracy but also in terms of specificity and sensitivity for both i) MCI-C vs MCI-NC and ii) MCI vs CN.

**Table 3:** Comparison of performance of the proposed method with existing works

	Method	Accuracy (%)	Sensitivity (%)	Specificity (%)
MCI-C vs. MCI-NC	Colliot et al. 2008 [13]	66	66	65
	Chupin et al. 2009 [15]		65	68
	Andrea Chincarini et.al., 2011 [16]	_	72	65
	Chong-Yaw Wee et.al., 2013 [19].	75.05	63.52	84.41
	Tong Tong et al., 2014 [20].	72	69	74
	Suk et al. 2014 [21]	72.42	36.70	90.98
	Liu et al. 2018 [24]	72.08	75.11	71.05
	**3D-DWT + LBP12**	0.8877	0.8916	0.9016
MCI vs. CN	Pennanen et al.2004 [12]	65.9	66.2	65.5
	Chupin et al. 2009 [15]		75	74
	Carlton Chu et al., 2011 [17]	67.3	_	_
	Chong-Yaw Wee et.al., 2013 [19].	92.33	83.55	83.95
	Suk et al. 2014 [21]	84.24	99.58	53.79
	Ahmed et al. 2015 [23]	78.22	70.73	83.34
	Khedher et al. 2015 [22]	80.27	73.51	82.70
	Liu et al. 2018 [24]	85.79	88.91	80.34
	**Proposed Model**	0.9031	0.9015	0.9022

Although each of the seven detailed components obtained with the application of 3D-DWT captures localized edges of different orientation, the number of features so obtained is quite large. Because of this, it requires large amount of memory and computation time to build a decision model. On the other hand, LBP-20 provides global distribution of patterns present in MRI volume with few features but omits finer details. The combination of 3D-DWT and LBP-20 brings advantages of the two methods. With the use of combination, the MRI volume is compactly represented in terms of distributions of different patterns from each of the seven detailed components obtained from 3D-DWT without omitting any relevant details. Hence, the combination of two methods provides better distinguishing power to differentiate two different types of MRI volumes. This is also reflected in our empirical analysis.

**Fig. 2:**
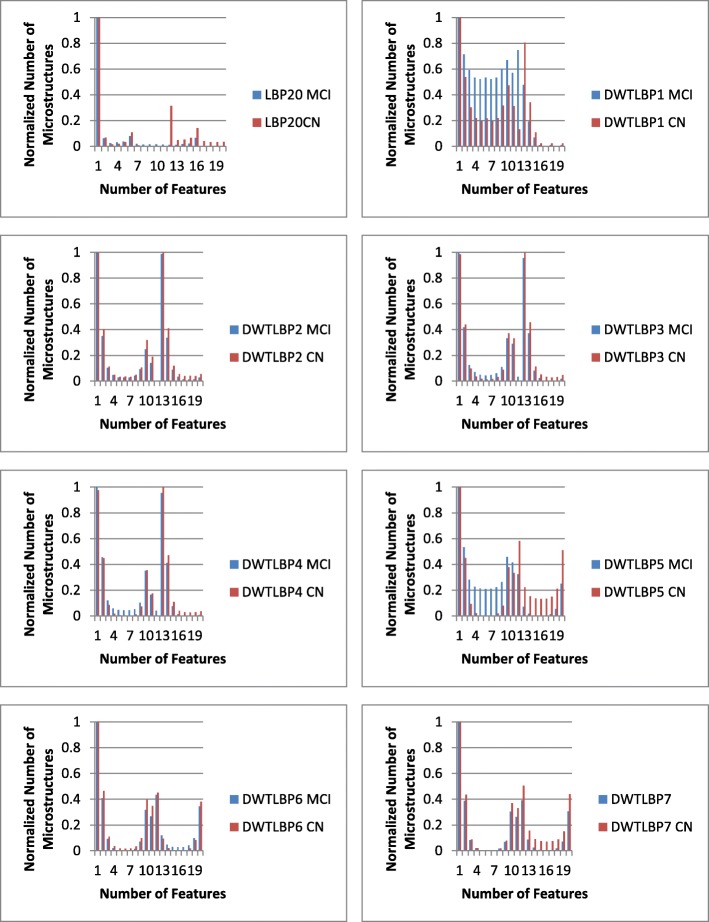
The comparison of the normalized features obtained with LBP-20 and 3D-DWT+ LBP-20: Here, DWT1, DWT2 etc. are the seven detailed components obtained using the 3D-DWT

To understand the better performance of the proposed technique in comparison to LBP-20, we initially normalized the individual features of both the methods i.e. LBP-20 and 3D-DWT + LBP-20. Subsequently the average value of individual feature is computed class wise, separately for both the methods. The comparison of the two methods to distinguish the two classes is shown in Fig. 2. The maximum difference between the values, in case of LBP-20 is 0.302 whereas the maximum difference between the values, in case of 3D-DWT + LBP20 is 0.613. This may be attributed to the ability of capturing more relevant patterns to distinguish the data of the two classes using the proposed method in comparison the LBP-20. It may also be noted that there are two or three peaks in most of the graphs, indicating that the corresponding bins may contribute to the classification of the two classes owing to a marked difference between the values of the two classes.

## Conclusion

In this paper, 3D variants of LBP called LBP-3D, LBP-309 and LBP-20 have been proposed. LBP-20 gives a better performance among the three variants of 3D-LBP and is combined with 3D-DWT in the proposed model, 3D-DWT + LBP-20, to extract relevant features from MRI for the classification between i) MCI-C and MCI-NC and ii) MCI and CN. The experimental results on publicly available ADNI datasets show that the proposed pipeline is quite effective to distinguish between the above-mentioned classes. It is also noted that the proposed combination of 3D-DWT and LBP-20 provides a better performance in comparison to 3D-LBP and its variants and it also performs better as compared to 3D-DWT in terms of specificity, accuracy and sensitivity. Also, the proposed method provides a superior performance with lesser number of features in comparison to the existing methods. This is attributed to the representation of MRI volume in terms of relevant and compact features, which are obtained with the application of LBP-20 on each of the seven detailed components of 3D.

In the future, the proposed method will be extended for multi-class classification. Also, in the future works, multivariate methods will be used for feature selection. Moreover, the analysis will be extended for other methods capable of finding out a smaller subset of relevant features which are more discriminative of the above-mentioned classes. As per Antoine Marie Jean-Baptiste Roger “*It is only with the heart that one can see rightly; what is essential is invisible to the eye.”* The above investigation suggests that *“heart”* can be replaced by *“pertinent feature extraction.”*

## Data Availability

http://adni.loni.usc.edu/data-samples/access-data/

## Abbreviations

AD: Alzheimer’s Disease
ADNI: Alzheimer’s DiseaseNeuroimaging Initiative
CN: Controls
CSF: Cerebro Spinal Fluid
CWT: Continuous Wavelet Transform
DWT: Discrete Wavelet Transform
FDR: Fisher Discriminant ratio
f-MRI: Functional-Magnetic Resonance Imaging
FT: Fourier-Transform
GLCM: Gray Level Co-occurrence Matrices
GM: Gray Matter
IP: Image processing
LBP: Local Binary Pattern
MCI: Mild Cognitive Impairment
MCI-C: Mild Cognitive Impairments Converts
MCI-NC: Mild Cognitive ImpairmentsNon-Converts
ML: Machine Learning
PET: Positron Emission Tomography
ROI: Region of Interest
s-MRI: Structural-Magnetic Resonance Imaging
SPM: Statistical Parametric Mapping
STFT: Short-Time-Fourier-Transform
WM: White Matter
WT: Wavelet Transform
